# Common genetic variation in the autoimmune regulator (*AIRE*) locus is associated with autoimmune Addison’s disease in Sweden

**DOI:** 10.1038/s41598-018-26842-2

**Published:** 2018-05-30

**Authors:** Daniel Eriksson, Matteo Bianchi, Nils Landegren, Frida Dalin, Jakob Skov, Lina Hultin-Rosenberg, Argyri Mathioudaki, Jessika Nordin, Åsa Hallgren, Göran Andersson, Karolina Tandre, Solbritt Rantapää Dahlqvist, Peter Söderkvist, Lars Rönnblom, Anna-Lena Hulting, Jeanette Wahlberg, Per Dahlqvist, Olov Ekwall, Jennifer R. S. Meadows, Kerstin Lindblad-Toh, Sophie Bensing, Gerli Rosengren Pielberg, Olle Kämpe

**Affiliations:** 1 Department of Medicine (Solna), Center for Molecular Medicine, Karolinska Institutet, Stockholm, Sweden; 2 Department of Endocrinology, Metabolism and Diabetes Karolinska University Hospital, Stockholm, Sweden; 3 Science for Life Laboratory, Department of Medical Biochemistry and Microbiology, Uppsala University, Uppsala, Sweden; 4 Science for Life Laboratory, Department of Medical Sciences, Uppsala University, Uppsala, Sweden; 5 Department of Molecular Medicine and Surgery, Karolinska Institutet, Stockholm, Sweden; 6 Department of Animal Breeding and Genetics, Swedish University of Agricultural Sciences, Uppsala, Sweden; 7 Department of Public Health and Clinical Medicine, Umeå University, Umeå, Sweden; 8 Department of Clinical and Experimental Medicine, Linköping University, Linköping, Sweden; 9 Department of Endocrinology, Linköping University, Linköping, Sweden; 10 Department of Medical and Health Sciences, Linköping University, Linköping, Sweden; 11 Department of Pediatrics, Institute of Clinical Sciences, Sahlgrenska Academy, University of Gothenburg, Gothenburg, Sweden; 12 Department of Rheumatology and Inflammation Research, Institute of Medicine, Sahlgrenska Academy, University of Gothenburg, Gothenburg, Sweden; 13 Broad Institute of MIT and Harvard, Cambridge, Massachusetts, United States of America; 14 K.G. Jebsen Center for Autoimmune Diseases, Bergen, Norway

## Abstract

Autoimmune Addison’s disease (AAD) is the predominating cause of primary adrenal failure. Despite its high heritability, the rarity of disease has long made candidate-gene studies the only feasible methodology for genetic studies. Here we conducted a comprehensive reinvestigation of suggested AAD risk loci and more than 1800 candidate genes with associated regulatory elements in 479 patients with AAD and 2394 controls. Our analysis enabled us to replicate many risk variants, but several other previously suggested risk variants failed confirmation. By exploring the full set of 1800 candidate genes, we further identified common variation in the *autoimmune regulator* (*AIRE*) as a novel risk locus associated to sporadic AAD in our study. Our findings not only confirm that multiple loci are associated with disease risk, but also show to what extent the multiple risk loci jointly associate to AAD. In total, risk loci discovered to date only explain about 7% of variance in liability to AAD in our study population.

## Introduction

The predominating cause of primary adrenal insufficiency is the autoimmune destruction of the adrenal cortex, known as autoimmune Addison’s disease (AAD)^1^. Affected patients suffer from loss of the essential adrenal hormones cortisol and aldosterone^2^. Although the symptoms typically develop slowly beginning with increasing fatigue, hyperpigmentation and weight loss, patients often present with acute adrenal insufficiency with abdominal pain, nausea and vomiting^1,3^. Prompt diagnosis and steroid replacement therapy is essential in order to avoid the otherwise fatal course of disease^1^. Autoantibodies targeting the adrenal enzyme 21-hydroxylase are highly specific diagnostic markers, and also confirm an autoimmune pathoaetiology^1,4,5^. The underlying causes of AAD are largely unknown, although the strong heritability and clear phenotype are incentives to genetic studies^6^.

AAD has a reported prevalence of 87–221 per million in European countries and the rarity of the disease has long rendered extensive genetic association studies unfeasible^7–14^. Most patients with AAD develop additional tissue-specific autoimmune diseases such as type 1 diabetes and autoimmune thyroid disease, both of which have been studied in genome-wide association studies^15^. Many risk genes that were identified for common autoimmune diseases have subsequently also been investigated via candidate-gene studies in AAD, and in this way, genes such as *HLA-DRB1* and *CTLA4* have been found to be associated with AAD^16–20^. Candidate-gene studies have also connected AAD with *PTPN22*^21^ and *CLEC16A*^22,23^ that are risk loci in several autoimmune diseases with complex inheritance.

AAD does not display a Mendelian inheritance pattern and the trait is considered complex^2^. However, in a small subset of patients, AAD appears as a component of the monogenic syndrome Autoimmune Polyglandular Syndrome type 1 (APS1) (Online Mendelian Inheritance in Man, # 240300). APS1 is caused by mutations in the autoimmune regulator gene (*AIRE*). The three main components of APS1 are AAD, chronic mucocutaneous candidiasis, and hypoparathyroidism, two of which are required for the clinical diagnosis^24,25^. The vast majority of patients with APS1 display autoantibodies against interferon-α, interferon-ω, and interleukin-22^26–29^. These biomarkers are highly specific for APS1 and can be used for identifying undiagnosed APS1 patients among patients with AAD^30^. In addition to recessively inherited APS1, dominant missense mutations in *AIRE* have also been more recently described^31–35^. Carriers of dominant *AIRE* mutations typically present a less severe phenotype with a later onset. Overall, APS1 accounts for only a minor fraction of all cases with AAD^30^.

We recently reported a targeted sequencing study that enrolled a large case group of 479 AAD patients from the Swedish Addison Register (SAR) and 1394 healthy controls; the SAR-Seq study^36^. Exons and regulatory regions of 1853 genes of interest were covered, enabling novel discoveries in the *BACH2* locus with genome-wide significance^36^. To date, this has been the most comprehensive genetic study of AAD. An important advantage of studies in rare diseases is that the general population is a sensible control group in genetic case-control studies. The SweGen Variant Frequency Database (1kSWE) harbours whole-genome variant frequencies from 1000 Swedish individuals^37^. Since no disease information has been collected, the 1kSWE represents a cross-section of the Swedish population rather than a set of healthy individuals. Still, with an AAD prevalence ranging from 87 to 221 per million in European studies^7–14^, the probability of including a significant number of AAD cases among a thousand Swedes is negligible (see Supplementary Table 1). Therefore, adding the allele counts from 1000 additional Swedish genomes could contribute to a more sensitive detection of disease risk loci with small effect sizes.

Here we present a study combining multiple AAD risk loci to evaluate their additive effects on disease risk and the age of disease onset. To expand the original SAR-Seq dataset and increase the number of detected variants severalfold, we imputed additional genotypes using haplotypes from the international 1000 genomes project^38^. With imputation and additional controls from the 1kSWE, our analysis could replicate several previous associations. By exploring potentially novel risk loci, we could also associate common variants in the *AIRE* gene to sporadic AAD.

## Results

### Imputation expands the dataset threefold

To date, the SAR-Seq study has generated the most comprehensive dataset on AAD risk loci. With imputation, we expanded the original data with three times as many common single-nucleotide polymorphisms (SNPs)^36^. By including allele counts from the 1kSWE^37^, we were able to compare allele frequencies from 479 AAD cases and 2394 controls (see Supplementary Figs S1 and S2). We set the study-wide statistical significance level to 1.2 × 10^−6^, a Bonferroni correction from α 0.05 for the number of independent variants (r^2^ < 0.8)^39^. We then applied the Cochran-Armitage test for trend to associate risk alleles with AAD and examined *P* value distributions using quantile–quantile plots (see Supplementary Fig. S3). Both the SAR-Seq subjects and the subjects in 1kSWE had been thoroughly investigated with principal component analysis to exclude non-European samples. Nevertheless, we adjusted the overdispersion of the test statistic (λ = 1.19) with genomic control to normalize λ before further analyses (see Supplementary Fig. S3).

### *CTLA4*, *BACH2*, *PTPN22* and *CLEC16A* are replicated risk loci in AAD

AAD has been associated with several genes, some of which have never been replicated since their first discovery in a candidate-gene study. In our previous targeted sequencing study, we systematically included genes that had previously been linked with AAD or other autoimmune disorders^36^. Our new imputed dataset contained 15 SNPs outside of the HLA region implicated in AAD in previous studies and allowed us to reinvestigate those suggested loci (Table 1). Except for *BACH2*, we were underpowered to associate the tabulated SNPs with AAD at our explorative study-wide significance level (*P* < 1.2 × 10^−6^). However, a number of these SNPs would have been significant in a candidate-gene study with just the few variants tested. Therefore, we regarded SNPs as replicated if they passed the nominal significance level (α 0.05) Bonferroni corrected for the 15 SNPs investigated (Table 1). At this level of significance (*P* value < 3.3 × 10^−3^), we replicated the well-established associations of *PTPN22*^21^, *CTLA4*^19,20,40,41^, and *CLEC16A*^22,23^. *BACH2* had already been associated with AAD in this case group^36^, but we could impute and confirm the SNP reported by Pazderska*et al*.^42^. However, the association to variants in *PD-L1*^43^, *GATA3*^44^, and *CYP27B1*^44^ could not be replicated even at the nominal significance level.

**Table 1:** Summary of allele frequencies and associations for variants outside the HLA region known or suggested to be associated with autoimmune Addison’s disease in previous studies.

Locus	Variant	Chr:Position^a^	Minor Allele Frequency		Association	
			SAR-Seq Cases	All Controls	OR^b^	*P* value^c^
*PTPN22*	rs2476601-A	1:114377568	0.18	0.12	1.52 (1.26–1.84)	6.1 × 10^−5*^
*CTLA4*	rs231806-C	2:204709349	0.30	0.36	0.74 (0.64–0.87)	5.8 × 10^−4*^
*CTLA4*	rs231775-G	2:204732714	0.53	0.46	1.09 (0.95–1.25)	5.5 × 10^−5*^
*CTLA4*	rs11571302-T	2:204742934	0.34	0.41	0.77 (0.66–0.89)	1.6 × 10^−3*^
*CTLA4*	rs7565213-A	2:204743409	0.34	0.41	0.77 (0.66–0.89)	1.6 × 10^−3*^
*CTLA4*	rs11571297-C	2:204745003	0.36	0.42	0.77 (0.66–0.90)	2.4 × 10^−3*^
*BACH2*	rs3757247-T	6:90957463	0.55	0.40	1.20 (1.04–1.38)	3.4 × 10^−15*^
*BACH2*	rs62408233-A	6:90976609	0.46	0.30	2.04 (1.77–2.36)	1.8 × 10^−19*^
*PD-L1*	rs1411262-T	9:5459419	0.22	0.25	0.85 (0.71–1.00)	0.07
*GATA3*	rs3802604-G	10:8102272	0.35	0.37	0.93 (0.80-1.08)	0.37
*GATA3*	rs569421-C	10:8108592	0.23	0.23	0.98 (0.83–1.16)	0.86
*CYP27B1*	rs4646536-G	12:58157988	0.35	0.37	0.94 (0.81-1.09)	0.44
*CYP27B1*	rs10877012-T	12:58162085	0.35	0.37	0.93 (0.80–1.07)	0.34
*CYP27B1*	rs703842-G	12:58162739	0.35	0.37	0.92 (0.79-1.06)	0.30
*CLEC16A*	rs12917716-C	16:11189148	0.35	0.41	0.78 (0.67–0.91)	2.3 × 10^−3*^

^a^All positions are given on reference genome hg19.

^b^The odds ratio (OR) represents the effect of the minor allele on the odds of observing AAD, calculated across SAR-Seq cases and all controls.

^c^The presented *P* values are adjusted with genomic control. ^*^Asterisk marks statistical significance after Bonferroni correction for the 15 investigated variants (*P* < 3.3 × 10^−3^).

### Common genetic variation in *AIRE* is associated to AAD

**Figure 1:**
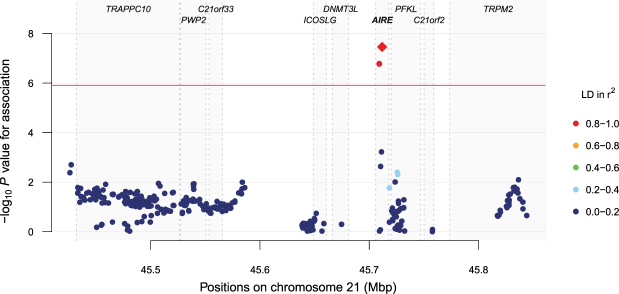
Association between genetic variants and autoimmune Addison’s disease at the 21q22.3 locus. *P* values are calculated with a Cochran-Armitage test for trend, adjusted with genomic control and plotted as the negative log_10_. The horizontal red line denotes the level of study-wide statistical significance, 1.2 × 10^−6^. Variants are coloured according to their correlation (r^2^) with the top SNP (red diamond, rs9983695), which has a *P* value of 3.5 × 10^−8^. Due to adjacent positions for three of the associated variants, their red data points overlap and only one red point is distinguishable in the figure in addition to the red diamond. The approximate location of the genes is indicated by gene names at the top of the plot and gray boxes in the background.

Our survey of the known risk loci consistently replicated several of the well-established associations. We next investigated the full range of variants detected in our array of captured exons, introns, and regulatory regions. Besides the strong peaks of association on chromosome 6, representing the HLA complex and the *BACH2* locus, we detected association to a novel locus on chromosome 21q22.3 (see Supplementary Fig. S4). A detailed description of the associated SNPs in the novel locus is presented in Table 2. The four tabulated SNPs were all study-wide significant and the top SNP exceeded the traditional cut off for genome-wide significance, 5 × 10^−8^ (rs9983695-C, OR = 0.37 (0.27–0.52), *P* = 2.1 × 10^−8^, MAF 0.04/0.11 in cases/controls). A closer look at the region revealed that all four SNPs were in strong linkage disequilibrium and located within the *AIRE* gene (Fig. 1, see Supplementary Fig. S5). The four associated SNPs were all located in introns 5 and 8, but did not co-localize with any known transcription factor binding sites (see Supplementary Fig. S6)^45,46^. For the newly associated SNPs in *AIRE*, the minor allele frequencies (MAF) were congruent across our control groups: SAR-Seq controls (10–11%) and the 1kSWE (10%). MAFs were also consistent with the European population of the international 1000 genomes project (11%), and the gnomAD non-Finnish European population (10–11%) (see Supplementary Table S2)^38,47^. To confirm the significantly lower MAF in our AAD cases, we regenotyped the samples using an alternative method. Using single base primer extension of the top associated SNP in *AIRE* (rs9983695), genotypes were identical to the genotypes obtained from sequencing data. We could therefore confirm that the MAF in cases was indeed 4%.

**Table 2:** Single-nucleotide variants at the *AIRE* locus associated with autoimmune Addison’s disease in single-variant association analysis.

Locus	Variant	Position^a^	Minor/Major Allele	Minor Allele Frequency				Association	
				AAD-Seq Cases	AAD-Seq Controls	1kSWE Controls	All Controls	OR^b^	*P* value^c^
*AIRE*	rs2075875	45709141	C/T	0.04	0.10	0.10	0.10	0.40 (0.29–0.55)	1.7 × 10^−7^
*AIRE*	rs2075876	45709153	A/G	0.04	0.10	0.10	0.10	0.39 (0.28–0.55)	1.6 × 10^−7^
*AIRE*	rs62220374	45709171	G/C	0.04	0.10	0.10	0.10	0.40 (0.29–0.55)	1.7 × 10^−7^
*AIRE*	rs9983695	45711808	C/T	0.04	0.11	0.10	0.11	0.37 (0.27–0.52)	3.5 × 10^−8^

^a^All positions are given on chromosome 21 (hg19).

^b^The odds ratio (OR) represents the effect of the minor allele on the odds of observing AAD, calculated across AAD-Seq cases and all controls.

^c^The presented *P* values are adjusted with genomic control.

*AIRE* mutations are well-established causes of monogenic Addison’s disease when it appears as a component in APS1, and thus explain a minor fraction of AAD cases. However, using both clinical criteria, screening for cytokine autoantibodies, and *AIRE* gene sequencing, our case group had already been thoroughly screened for APS1 and all suspected patients excluded^30^. In line with that, we could not detect any carriers of known dominant or novel protein-altering mutations in *AIRE*, but identified a single heterozygous carrier of the recessive R257* (rs121434254-T) variant. In conclusion, common genetic variation in *AIRE* appeared to be reliably associated to AAD even after strict removal of cases with suspected APS1.

### Multiple AAD risk loci display additive effects

**Figure 2:**
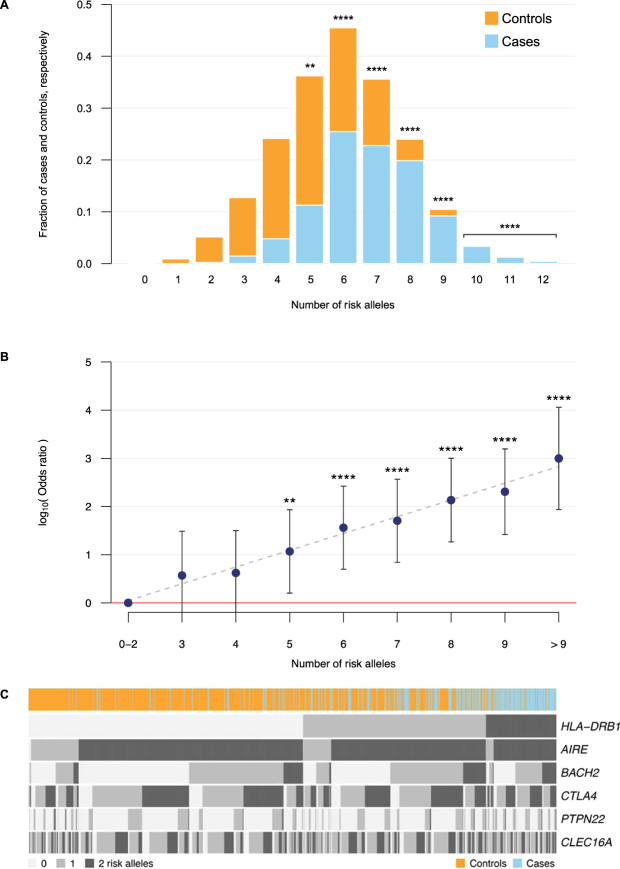
The number of risk alleles in cases and controls across six associated risk loci. The included risk alleles are rs9983695-T in *AIRE*, rs62408233-A in *BACH2*, rs231806-G in *CTLA4*, rs2476601-A in *PTPN22*, rs12917716-G in *CLEC16A*, and 03:01, 04:04, and 04:03 in HLA-*DRB1*. In panel (A), the top bars (orange) represent the fraction of controls and add up to 1, whereas the bottom bars (blue) indicate fraction of cases and also add up to 1. Panel (B) shows the log-transformed odds of AAD dependent on the number of risk alleles. The red line displays the level of the reference group, subjects carrying 0 to 2 risk alleles. The linear model, log_10_(Odds ratio) = 0.35*x*  – 0.65, where *x* is the number of additional risk alleles, is depicted by a dashed grey line and demonstrates that the odds ratio is more than doubled with every additional AAD risk allele (R^2^ = 0.97). Vertical bars indicate 95% confidence intervals for the point estimates. Panel (C) shows in detail the number of risk alleles across six risk loci and SAR-Seq subjects. Each column represents a subject, and the top bar displays orange colour for controls and blue for cases. The heatmap shows light, medium and dark gray for 0, 1 and 2 risk alleles, respectively. Risk loci have been ordered by descending odds ratio, and subjects sequentially sorted to facilitate interpretation. Furthest to the left, are all subjects with 0 risk alleles, and furthest to the right, all subjects with 12 risk alleles. In panels (A) and (B), **indicates *P* value < 0.01 and ****P < 0.0001, compared to the odds in the group of samples carrying 0 to 2 risk alleles. Samples with 10 or more risk alleles are grouped together in statistical testing.

Complex diseases are typically influenced by a large number of genes^48^. To study the joint effects of AAD risk variants, we investigated the distribution of risk variants among cases and controls. At six loci, we counted the number of risk alleles carried in each of the 479 cases and 1394 controls from the SAR-Seq study. We included the confirmed risk alleles rs62408233-A in *BACH2*, rs231806-G in *CTLA4*, rs2476601-A in *PTPN22*, rs12917716-G in *CLEC16A*, and the alleles 03:01, 04:04, and 04:03 in *HLA*-*DRB1*. In addition, we included the newly identified rs9983695-T in *AIRE*. When we stratified the cases and controls by their number of risk alleles, we observed that the fraction of cases tends to rise with an increasing number of risk alleles (Fig. 2A). It was intriguing to note the low proportion of cases emerging in strata with less than three risk alleles, and at the other extreme, the high proportion of cases among subjects with more than nine risk alleles. With reference to subjects with less than three risk alleles, we could estimate with a linear model that the odds ratio for AAD more than doubles with every additional AAD risk allele (Fig. 2B). For instance, the odds for disease is 200 (95% CI: 26–2000) times higher in cases with 9 risk alleles, compared to cases with 0, 1 or 2 risk alleles (see Supplementary Table S3). For subjects with 10, 11 or 12 risk alleles, the odds of disease are even higher. In order to visualize the distribution of risk alleles in detail, we constructed a heatmap showing the number of risk alleles each study sample carried in the same 6 risk loci (Fig. 2C). *HLA-DRB1* risk alleles are known to confer the highest risk, especially when inherited in homozygous form, or heterozygous with particular *HLA-DRB1* risk alleles^18,36^. But even within different HLA strata, effect from the other risk loci was evident, perfectly in line with joint effects from independent loci (Fig. 2C).

In genetic interaction, risk alleles at different loci act in synergy, producing a joint effect exceeding the sum of their separate effects^49^. To investigate whether AAD risk alleles displayed any statistical interaction, we selected the significant risk alleles in Table 1, 03:01, 04:04 and 04:03 in *HLA*-*DRB1*, and applied a logistic regression model with interaction terms, as implemented in the PLINK software and with default parameterization. Beyond the additive, however, no synergistic effects on disease risk could be detected (see Supplementary Table S4). We next assessed whether a subject’s age at disease onset was dependent on their combined risk allele load (see Supplementary Fig. S7). On average, subjects with more than nine risk alleles acquired their AAD more than eight years earlier than subjects with less than five risk alleles (*P* = 0.018, 95% CI: −15.8 to −1.5) (see Supplementary Table S5). However, this difference was outweighed by the variance within each stratum. No single loci could be associated to age of onset and the top SNP (rs1569495-C on chromosome 22, *P* = 5.0 × 10^−6^, MAF 0.2356) had a false discovery rate of 23% (see Supplementary Fig. S7).

### Only a minor fraction of heritability is explained

**Figure 3:**
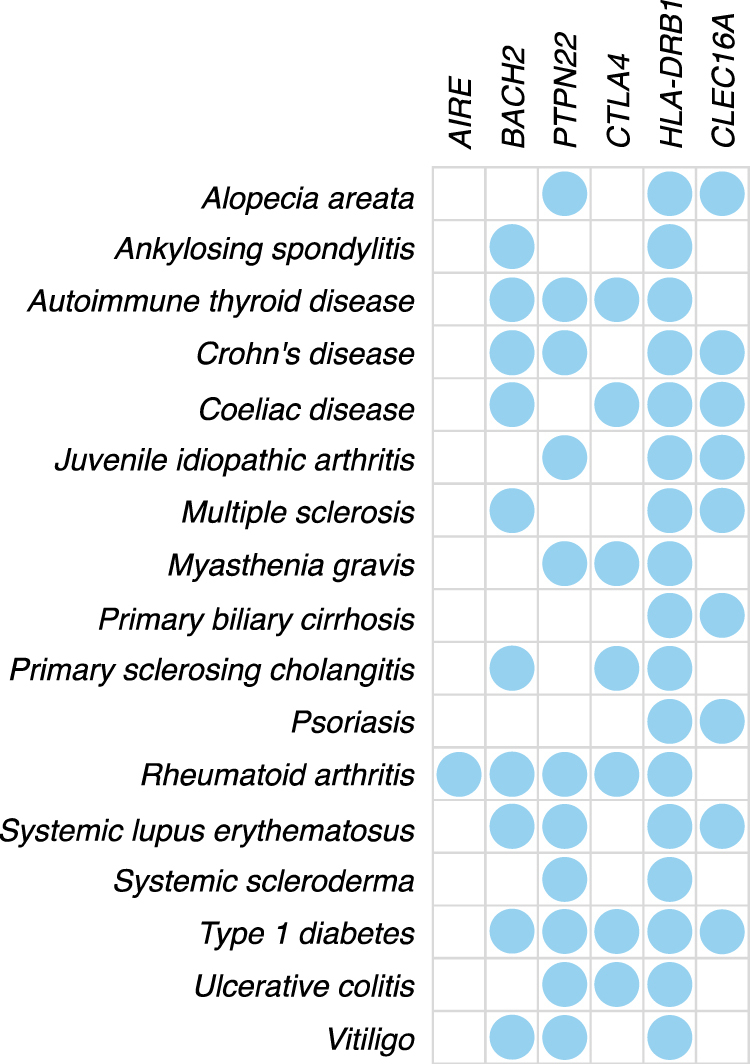
Loci implicated in autoimmune Addison’s disease and their associations to other autoimmune diseases in genome-wide association studies. GWAS and Immunochip associations from the NHGRI-EBI catalog of published GWAS and *immunobase*.*org*. Blue dots indicate genome-wide significant associations for the diseases and the loci.

Several organ-specific autoimmune diseases such as type 1 diabetes and autoimmune thyroid disease, are co-occurring in families and individuals, reflecting their shared risk factors^15^. AAD has many risk loci in common with other autoimmune diseases (Fig. 3)^50^. Yet, the SNPs significantly associated to AAD and covered in this study explain a mere ~7% (range 5–9% depending on prevalence, SE range 0.7–1%) of the variance in liability in our study population. With all independent SNPs in our final dataset, including SNPs not associated to AAD, 28% (SE 4%) of liability is explained. Hence, most of disease heritability remains to be explained in future studies.

In the past, sample sizes have been large enough to make successful candidate-gene studies in AAD, yet too small to discover the same genes in a GWAS setting. With increasing sizes of AAD sample collections, we wanted to address the question whether we have already reached the sample sizes required to discover novel risk loci in a GWAS. We therefore estimated the number of cases and controls required to discover risk loci with allele frequencies and odds ratios comparable to the well-established risk loci given a traditional genome-wide significance level, 5.0 × 10^−8^. Established risk alleles in *CTLA4* and *PTPN22* do not require any additional confirmation but their frequencies and impacts can exemplify what to expect of AAD risk alleles. Assuming five healthy controls for every AAD case, around 2000 cases would suffice to discover risk alleles with properties comparable to *PTPN22* and *CTLA4* in our Swedish dataset, each with 80% statistical power (see Supplementary Table S6). To discover risk loci with smaller effect sizes, in the range of *CLEC16A*, would require approximately 3000 cases. The estimated sample sizes and alternative proportions of cases and controls are presented in Supplementary Table S6.

## Discussion

The cause of autoimmune Addison’s disease is largely unknown but the strong heritability in our Swedish population enables insight through genetic studies^6^. Although several risk loci have been studied one by one, they have not been comprehensively investigated together. In this study, we simultaneously investigated established and suggested risk loci, and assessed both their independent and joint effects.

We started this study by revisiting several risk variants that had been established or proposed in previous case-control studies. For the risk variants in *PTPN22* and *CLEC16A*, we confirmed the odds ratios reported in previous studies^21–23,51^. Likewise, for *CTLA4*, we could replicate findings for non-coding variants, both upstream and downstream of the gene^20^. The common missense mutation in *CTLA4* exon 1 (rs231775, c.49 A > G, p.Thr17Ala) also showed association to AAD, but with a weaker odds ratio compared to the non-coding SNPs^19,41^. We could not, however, replicate the associations to *PD-L1*^43^, *GATA3*^44^ or *CYP27B1*^44^ despite covering the suggested loci and previously associated variants.

We next explored our full range of genetic variants and identified *AIRE* as a novel risk locus in AAD with complex inheritance. Interestingly, recessive *AIRE* mutations cause APS1, of which AAD is a major component. Dominant *AIRE* mutations typically give rise to milder disease phenotypes with later onset compared to the recessive APS1, but can also present with AAD^31,33,34^. Family members carrying dominant *AIRE* mutations can acquire various autoimmune manifestations, some of which often co-occur with sporadic AAD^34^. These links between *AIRE* and AAD raise the question of whether the association we identified in *AIRE* could be due to the presence of undiagnosed APS1 patients in our case group. However, in addition to excluding all diagnosed cases of APS1, we took several measures to identify suspected APS1 patients prior to association testing. First, we sequenced the *AIRE* exons without detecting any known or novel protein-altering mutations indicative of APS1. We also excluded cases with cytokine autoantibodies targeting type 1 interferons α and ω, as well as IL-22, typical of recessive APS1 and also present in some cases with dominant APS1^34,52–55^. Furthermore, by excluding cases and controls with high degree of relatedness, we selected independent samples rather than families to avoid including cases of monogenic disease. It therefore appears unlikely that the identified association with *AIRE* was explained by undiagnosed cases of APS1.

Taken together, our results suggest that common genetic variation in the *AIRE* locus may influence the susceptibility to AAD, even in the absence of protein alteration. This is in contrast with a previous Norwegian study that investigated one of our top associated SNPs (rs2075876-A/G) in a smaller Addison’s disease case group (n = 311), without finding a disease association^56^. Hence, in the context of previous studies, our results do not infer a final relationship between AAD development and the studied SNPs, but again point to the *AIRE* locus as a factor in autoimmune disease, beyond recessive APS1. Future studies in additional AAD case groups are required to fully establish an association to disease, and functional studies to investigate how a potential causal relationship could be mediated. Since AAD risk loci may also differ between studies of different populations^44^, it would be especially valuable to replicate this novel finding in other populations.

It is not uncommon that monogenic and complex diseases with similar phenotypes share risk loci. For instance, *CTLA4* is mutated in the monogenic autoimmune lymphoproliferative syndrome type V (OMIM #616100), which has bowel inflammation as an important component^57,58^, and is associated with sporadic forms of inflammatory bowel disease (IBD)^59^. Another example is the genes in the interleukin-10 pathway, in which rare mutations cause severe IBD in neonates (OMIM #613148), and common variation is linked to sporadic IBD^59,60^. Mutations in the *INS* (*insulin*) and *GLIS3* (*GLIS Family Zinc Finger 3*) genes cause monogenic neonatal diabetes (OMIM #606176, #610199), while common variations are linked to sporadic type 1 diabetes^61^. The *AIRE* gene, mutated in APS1, appears to harbor common variants associated to the risk of AAD in our Swedish case group. Common genetic variation in *AIRE* has previously been linked to rheumatoid arthritis in GWA studies in Japanese and Chinese Han populations^62,63^. However, it is worth noting that the risk of rheumatoid arthritis is linked to the allele rs2075876-A, whereas we associate AAD to the other allele, rs2075876-G. It may seem contradictory, but apparent shared risk loci and shared risk alleles can reflect different underlying causal variants or be influenced by other factors. The risk allele rs2075876-A has failed in validation studies of rheumatoid arthritis in European cohorts^64^.

Sporadic AAD is considered a complex trait where no single gene is strictly required and no single gene is sufficient to cause the disease. Therefore, we investigated the joint effects of the confirmed risk loci known today. It was striking to note the high proportion of cases in strata with more than nine AAD risk alleles. Since we only observed a few subjects with more than nine risk alleles in our case group, these combinations of alleles could only explain a fraction of the overall disease heritability. However, it was interesting to note that subjects carrying 12 risk alleles appear to have a large proportion of their genetic propensity of AAD explained by the investigated six loci. Looking in detail at which risk alleles were present in each of our subjects, the results were in line with independent segregation of risk alleles with additive effects. With the studied risk alleles, we found a subtle effect on the age of disease onset. The number of risk alleles was not a strong predictor, but the overall effect is clear: the higher the burden of risk alleles, the earlier the disease onset.

In total, the significant risk loci *HLA*, *AIRE*, *BACH2*, *CTLA4*, *PTPN22* and *CLEC16A* explained about 7% of variance in liability of AAD in our case group. Considering that the heritability statistic is dependent on the study population and the given environment, it is ill-suited for comparison between studies. It has, however, become clear that GWAS in autoimmune diseases tend to explain only a minor fraction of heritability, for instance 9% in studies of Graves’s disease and 16% in ulcerative colitis^65–67^. By including the full range of variants detected in our array of captured exons, introns, and regulatory regions, an additional 23% of liability in our case group was explained. Consequently, 77% (23/(7 + 23)) of SNP variation has remained undetected in our study because the effect sizes have been too small to pass our statistical significance threshold. The discrepancy between the 23% accounted for by all SNPs and the total disease heritability (>90%), suggests that additional genetic variation is left to discover outside of our investigated genomic regions and/or in other classes of genetic variation, for instance, insertions, deletions and copy number variations.

Prior to SAR-Seq, most genetic studies in AAD had been limited to candidate gene approaches^2^. These candidate genes were typically selected based on previous associations with autoimmune disorders. In fact, all currently known AAD risk loci are associated with other autoimmune diseases. This probably reflects how these loci were selected for investigation, but may also point towards a high degree of sharing of genetic risk factors in autoimmunity. However, as the number of Addison’s disease samples contained within biobanks continues to get larger, we have reached the time when hypothesis-free explorations of common genetic variation are feasible with reasonable statistical power. Our calculations show that around 2000 AAD cases would suffice to replicate many of the well-established risk loci with genome-wide significance. Considering that most of the genetics underlying AAD is still uncharted, even smaller case groups would provide opportunities for novel findings.

Even though the SweGen Variant Frequency Database had made allele counts public, access to individual genotypes was still pending at the time of analysis. Therefore, the main association testing was made without population substructure covariates. Rather, the hundreds of thousands of variants tested enabled correction of the test statistics by means of genomic control. Replication in other populations and larger case groups could strengthen the association to *AIRE* and will be a natural part of large-scale genetic studies investigating the whole genome of AAD patients. Characterized by high heritability in our Swedish population and high co-inheritance with other autoimmune diseases, AAD is an attractive subject for genetic studies of sporadic autoimmunity. Furthermore, as pointed out in this study, the rarity of Addison’s disease can paradoxically be an advantage in genetic studies, as more and more healthy individuals are genotyped worldwide, and the AAD diagnose can be made with high specificity.

## Methods

### Study subjects

This study included the final 479 AAD cases and 1394 controls previously described in detail in the SAR-Seq study^36^. In brief, all case subjects fulfilled diagnostic criteria for primary adrenal insufficiency^1^ and were required to test positive in two independent assays for 21-hydroxylase autoantibodies, performed at two separate laboratories. To focus the study on polygenic autoimmune Addison’s disease, case subjects where other causes of adrenal failure could be suspected were excluded; for example, APS1, adrenal metastases, adrenalectomy, congenital adrenal hyperplasia, hypogonadotropic hypogonadism, isolated ACTH deficiency, tuberculosis or adrenal failure secondary to corticosteroid treatment. Carriers of autoantibodies targeting the cytokines interferon ω, interferon α and/or interleukin 22 were excluded because undiagnosed APS1 could be suspected. SAR-Seq controls were included from blood donors (n = 848, Uppsala Bioresource) and directed sampling of healthy volunteers (n = 653). All subjects were carefully investigated with regards to ancestry and kinship, excluding non-European subjects and first-degree relatives. For the present study, allele counts from the SAR-Seq study and the 1kSWE were merged to augment the control population (see Supplementary Fig. S1)^37^.

All study subjects were collected in Sweden and gave their informed consent. The SAR-Seq study was performed in accordance with the Declaration of Helsinki and was approved by the local ethics committees 2008/296-31/2 in Stockholm, 2009/013 in Uppsala, 02-253 in Umeå, and 98110 in Linköping, Sweden.

### Sequencing of target genes

Targeted sequencing was described in detail in the SAR-Seq study^36^. In brief, a selection of 1853 genes was targeted by our custom designed SeqCap EZ Choice XL capture library (06266517001; Roche NimbleGen, Basel, Switzerland). Besides genes causing monogenic adrenal disease, genes associated with autoimmune disease in genome-wide association studies were systematically included. Exons, promoters and surrounding conserved elements were targeted^68^. DNA samples were sonicated to 400-bp fragments and sequenced with 100-bp paired-end reads using Illumina HiSeq2500 (Illumina, San Diego, CA, US).

### Bioinformatic processing of targeted sequencing data

The processing from sequencing reads to a high quality call set was comprehensively explained in the SAR-Seq study^36^. In essence, for each sample, sequencing reads were mapped to the hg19 human reference genome^69^. Samples with mean target coverage less than 10X were excluded. Variant calling was carried out using HaplotypeCaller (GATK 3.2.2) in gVCF mode, according to the procedures recommended for single samples all-sites calling [34, 35]. Final genotyping was performed collectively for all SAR-Seq samples with GenotypeGVCFs. Thereafter, only SNPs were considered in further analyses. Genotype calls with depth less than 8 reads or a genotype quality score less than 20, were excluded^70,71^. Remaining SNPs were assigned probability scores using the VariantRecalibrator (GATK 3.2.2) and filtered with sensitivity tranche level 99.0.

### Proportion of variance in liability explained by SNPs

In PLINK 1.9, SNPs with MAF larger than 1% were pruned such that no pairs of markers within a 150-variant window had a squared correlation stronger than 0.8^72^. GCTA (Genome-wide Complex Trait Analysis)^73^ v.0.93.9 was then used to estimate a genomic relationship matrix, and to perform a restricted maximum likelihood analysis, assuming an AAD prevalence ranging from 87 to 221 per million.

### Imputation, dataset merging and quality control

Imputation with IMPUTE2 version 2.3.2^74^ was performed using the full international 1000 genomes Phase 3 (October 2014), based on haplotypes obtained from 2504 individuals. To maximize imputation accuracy, no pre-phasing was used. Default and recommended settings were applied for effective population size (20 000) and the region size (5 × 10^6^ bp). Imputed genotypes with certainty less than 95% were flagged as missing, and subsequently positions with more than 20% missing calls were definitively excluded before merging the SAR-Seq dataset with allele counts from 1kSWE. The merging and quality control procedures are outlined in the supplementary flowchart, where cut-off values are specified in detail (see Supplementary Fig. S1). The merging with 1kSWE controls was done in R v3.2.1^75^. Biallelic SNPs overlapping the SAR-Seq dataset were extracted from the 1kSWE. Available quality control metrics were employed to stringently exclude low quality variants from 1kSWE (see Supplementary Fig. S2). Variants with differential missingness between SAR-Seq cases and controls were flagged for exclusion. Hardy-Weinberg equilibrium was tested both in each of the control sets separately, and subsequently in the merged control set. To further ensure a homogenous merged control set, variants significantly associated to either of the control groups in a Cochran-Armitage test for trend (*P* < 0.001) were flagged for exclusion. Variants with MAF less than 0.05 in the SAR-Seq dataset, the 1kSWE, or the merged dataset were flagged for exclusion, to increase power to associate more common variants. Variant filters were effectuated in parallel and many variants failed on more than one criterion. The number of independent SNPs in downstream analyses was calculated in PLINK 1.9 by pruning the dataset such that no pairs of variants within a 150-variant window were correlated (r^2^) more than 0.8^39,72,76^.

### Variant and gene annotation

The impacts of alleles were predicted using SnpEff 4.1 [40]. Curated epigenetic information for genomic positions was retrieved from HaploReg 4.1 and RegulomeDB^45,46^. Diseases that share potential risk loci with AAD were collected from the NHGRI-EBI catalog of published GWAS and *immunobase*.*org*^77,78^. Gene locations were extracted from the genome browser at UCSC, assembly hg19^79^.

### Validation genotyping

The validation genotyping was performed using single base primer extension and fluorescent polarization template dye incorporation. Signal intensities were detected in a Hidex Sense (Hidex, Turku, Finland) fluorescence absorbance reader, and genotypes were called with the AlleleCaller 4.0.0.1 software.

### Statistical methods

PLINK 1.9 was used for allele counts, differential missingness, conditional analysis in logistic regression, and merging with the imputed data^72,76^. Study-wide association was tested with a two-sided Cochran Armitage test for trend in R. Inflation factor λ was estimated from the median χ^2^ and used for genomic control^80^. LD structures were measured and visualized by squared correlation (r^2^) in Haploview^81^. Sample size calculation used the normal distribution without continuity correction as an approximation to the binomial distribution. Odds ratios from contingency tables, and *t*-tests for different number of risk alleles were calculated in GraphPad Prism v6.0 h. Linear models were calculated with the glm function in R. Screening for gene-gene interaction was performed using the epistasis command in PLINK 1.9, testing a logistic regression model for statistical interaction; ln (P(Y = case)/P(Y = control)) = β_0_ + β_1_g_A_ + β_2_g_B_ + β_3_g_A_g_B_. Proportions of alleles conferring risk versus protection were compared between control groups using χ^2^ test in R.

## Electronic supplementary material

Supplementary information
