# Supplementary material for: Common genetic variation in the autoimmune regulator (AIRE) locus is associated with autoimmune Addison’s disease in Sweden
Source: Sci Rep. 2018 May 30;8:8395. doi: 10.1038/s41598-018-26842-2 (PMC5976627; doi:10.1038/s41598-018-26842-2)
Supplement: Supplementary file 1 — Supplementary information [file 41598_2018_26842_MOESM1_ESM.docx]

Supplementary Information

**Common genetic variation in the autoimmune regulator (*AIRE*) locus is associated with autoimmune Addison’s disease in Sweden**

Daniel Eriksson^1, 2^ *, Matteo Bianchi^3^, Nils Landegren^1, 4^, Frida Dalin^1, 4^, Jakob Skov^5^, Lina Hultin-Rosenberg^3^, Argyri Mathioudaki^3^, Jessika Nordin^3^, Åsa Hallgren^1^, Göran Andersson^6^, Karolina Tandre^4^, Solbritt Rantapää Dahlqvist^7^, Peter Söderkvist^8^, Lars Rönnblom^4^, Anna-Lena Hulting^5^, Jeanette Wahlberg^8, 9, 10^, Per Dahlqvist^7^, Olov Ekwall^11, 12^, Jennifer R. S. Meadows^3^, Kerstin Lindblad-Toh^3, 13^, Sophie Bensing^2, 5^, Gerli Rosengren Pielberg^3^, Olle Kämpe^1, 2, 14^

^1^Dept. of Medicine (Solna), Center for Molecular Medicine, Karolinska Institutet, Stockholm, Sweden. ^2^Dept. of Endocrinology, Metabolism and Diabetes Karolinska University Hospital, Stockholm, Sweden. ^3^Science for Life Laboratory, Dept. of Medical Biochemistry and Microbiology, Uppsala University, Uppsala, Sweden. ^4^Science for Life Laboratory, Dept. of Medical Sciences, Uppsala University, Uppsala, Sweden. ^5^Dept. of Molecular Medicine and Surgery, Karolinska Institutet, Stockholm, Sweden. ^6^Dept. of Animal Breeding and Genetics, Swedish University of Agricultural Sciences, Uppsala, Sweden. ^7^Dept. of Public Health and Clinical Medicine, Umeå University, Umeå, Sweden. ^8^Dept. of Clinical and Experimental Medicine, Linköping University, Linköping, Sweden. ^9^Dept. of Endocrinology, Linköping University, Linköping, Sweden. ^10^Dept. of Medical and Health Sciences, Linköping University, Linköping, Sweden. ^11^Dept. of Pediatrics, Institute of Clinical Sciences, Sahlgrenska Academy, University of Gothenburg, Gothenburg, Sweden. ^12^Dept. of Rheumatology and Inflammation Research, Institute of Medicine, Sahlgrenska Academy, University of Gothenburg, Gothenburg. ^13^Broad Institute of MIT and Harvard, Cambridge, Massachusetts, United States of America. ^14^K.G. Jebsen Center for Autoimmune Disorders, Bergen, Norway.

**Corresponding author**

Daniel Eriksson, [daniel.eriksson@ki.se](mailto:nils.landegren@ki.se), Department of Medicine Solna, Karolinska Institute, Experimental endocrinology, Center for Molecular Medicine, L8:01, Karolinska University Hospital, SE-171 76 Stockholm.

Supplementary Information

| *Item* | *Page* |
| --- | --- |
| Table S1 | 3 |
| Figure S1 | 4 |
| Figure S2 | 5 |
| Figure S3 | 6 |
| Figure S4 | 7 |
| Figure S5 | 8 |
| Figure S6 | 8 |
| Table S2 | 9 |
| Table S3 | 10 |
| Table S4 | 10 |
| Figure S7 | 11 |
| Table S5 | 11 |
| Table S6 | 12 |
|  |  |

| **Table S1. The probability of including autoimmune Addison’s disease (AAD) cases among 1000 random subjects, depending on disease prevalence.** | | | | | | |
| --- | --- | --- | --- | --- | --- | --- |
|  |  | **Prevalence 87 per million** | |  | **Prevalence 221 per million** | |
| **AAD cases (*k*)** |  | **Probability of exactly *k* cases** | **Probability of *k* or fewer cases** |  | **Probability of exactly *k* cases** | **Probability of *k* or fewer cases** |
| 0 |  | 0.904833 | 0.904833 |  | 0.801697 | 0.801697 |
| 1 |  | 0.090492 | 0.995325 |  | 0.177214 | 0.978911 |
| 2 |  | 0.004521 | 0.999846 |  | 0.019567 | 0.998478 |
| 3 |  | 0.000150 | 0.999996 |  | 0.001439 | 0.999917 |
| 4 |  | 0.000004 | 1.000000 |  | 0.000079 | 0.999996 |
| 5 |  | 0.000000 | 1.000000 |  | 0.000003 | 1.000000 |
| 6 |  | 0.000000 | 1.000000 |  | 0.000000 | 1.000000 |
| 7 |  | 0.000000 | 1.000000 |  | 0.000000 | 1.000000 |
| 8 |  | 0.000000 | 1.000000 |  | 0.000000 | 1.000000 |
| 9 |  | 0.000000 | 1.000000 |  | 0.000000 | 1.000000 |
| 10 |  | 0.000000 | 1.000000 |  | 0.000000 | 1.000000 |
|  |  |  |  |  |  |  |

**Figure S1. In total, after strict quality control, imputation expanded the original SAR-Seq dataset with three times as many common variants.**

The flowchart visualizes the fusion of control datasets and major quality control steps. The number of variants flagged for exclusion by each filter is given within parentheses. Since variant filters were applied in parallel and many variants failed on more than one criterion, the sum of the figures within parentheses exceeds the total number of excluded variants. Finally, stringent quality control excluded 43% of overlapping variants and resulted in 392 360 common SNPs available for association testing (MAF ≥ 5%).

| **A** | **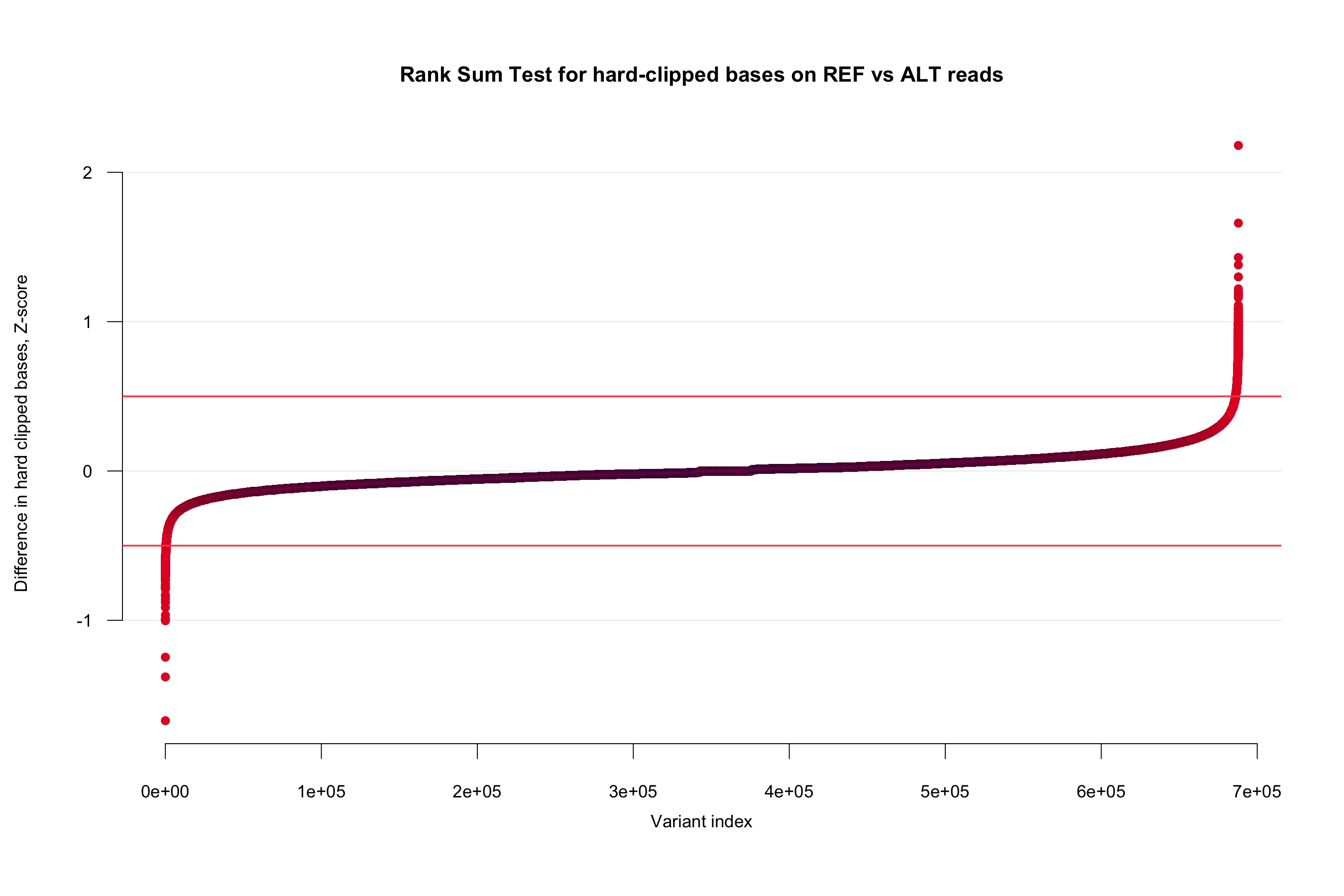** | **B** | **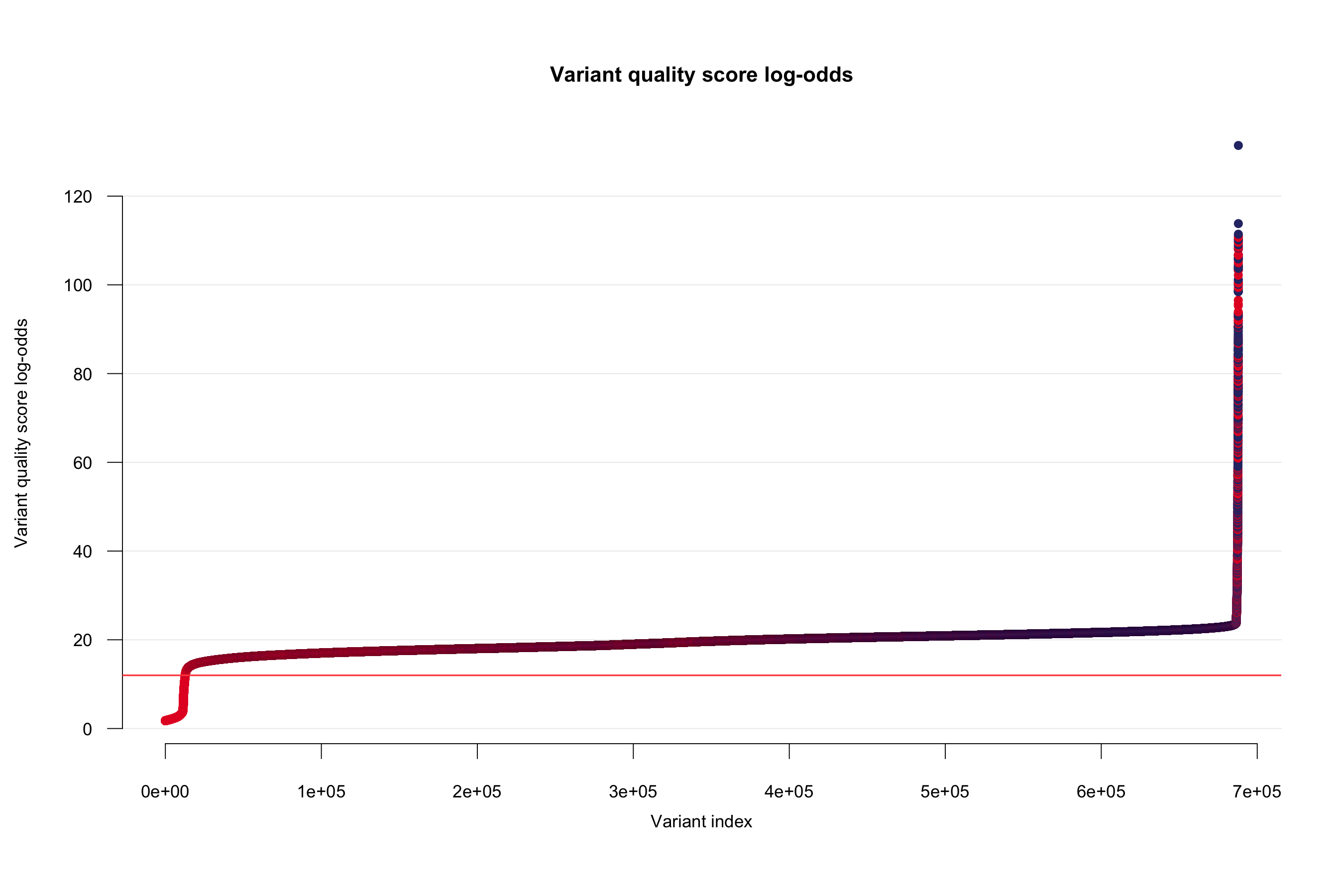** |
| --- | --- | --- | --- |
| **C** | **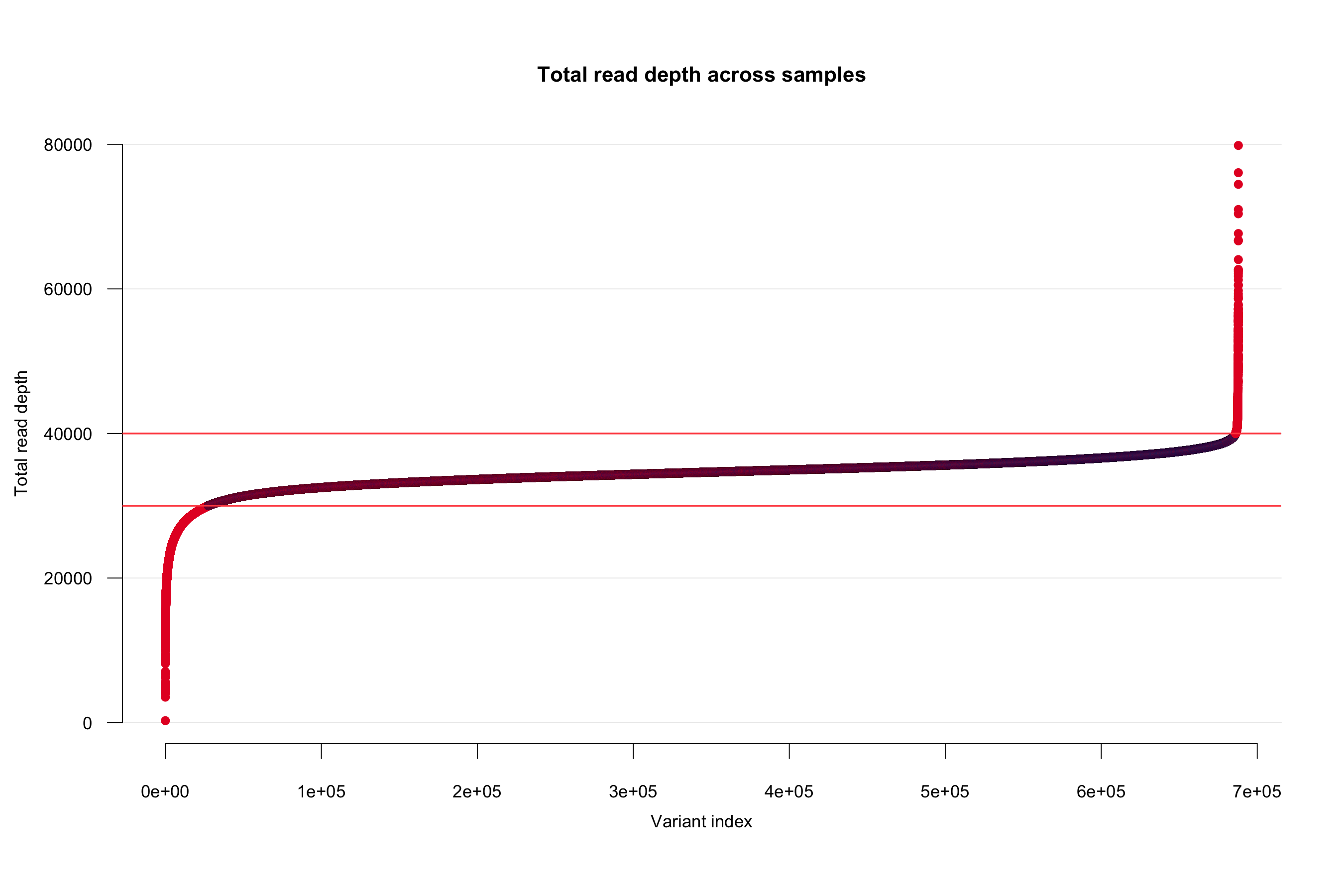** | **D** | **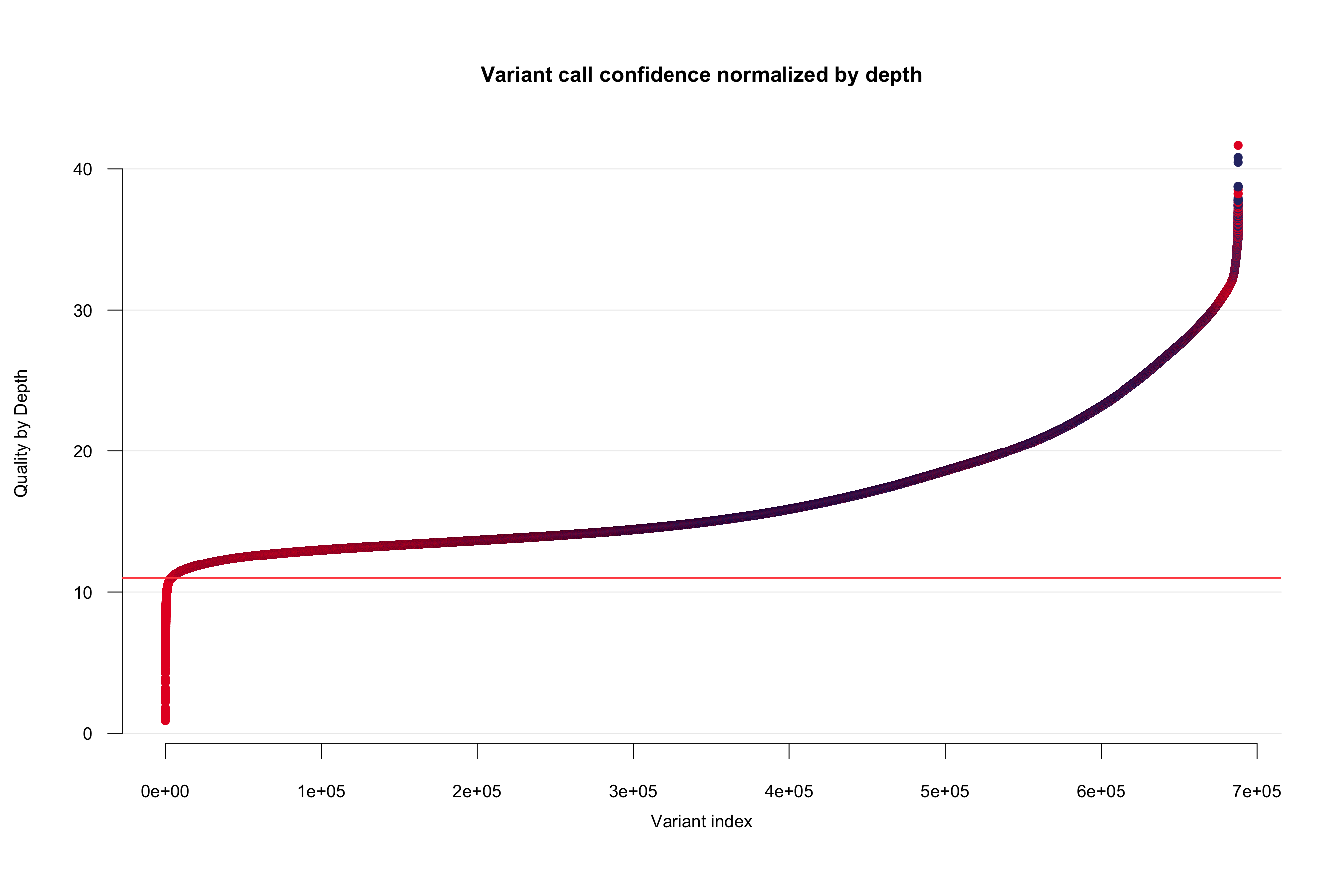** |
| **E** | **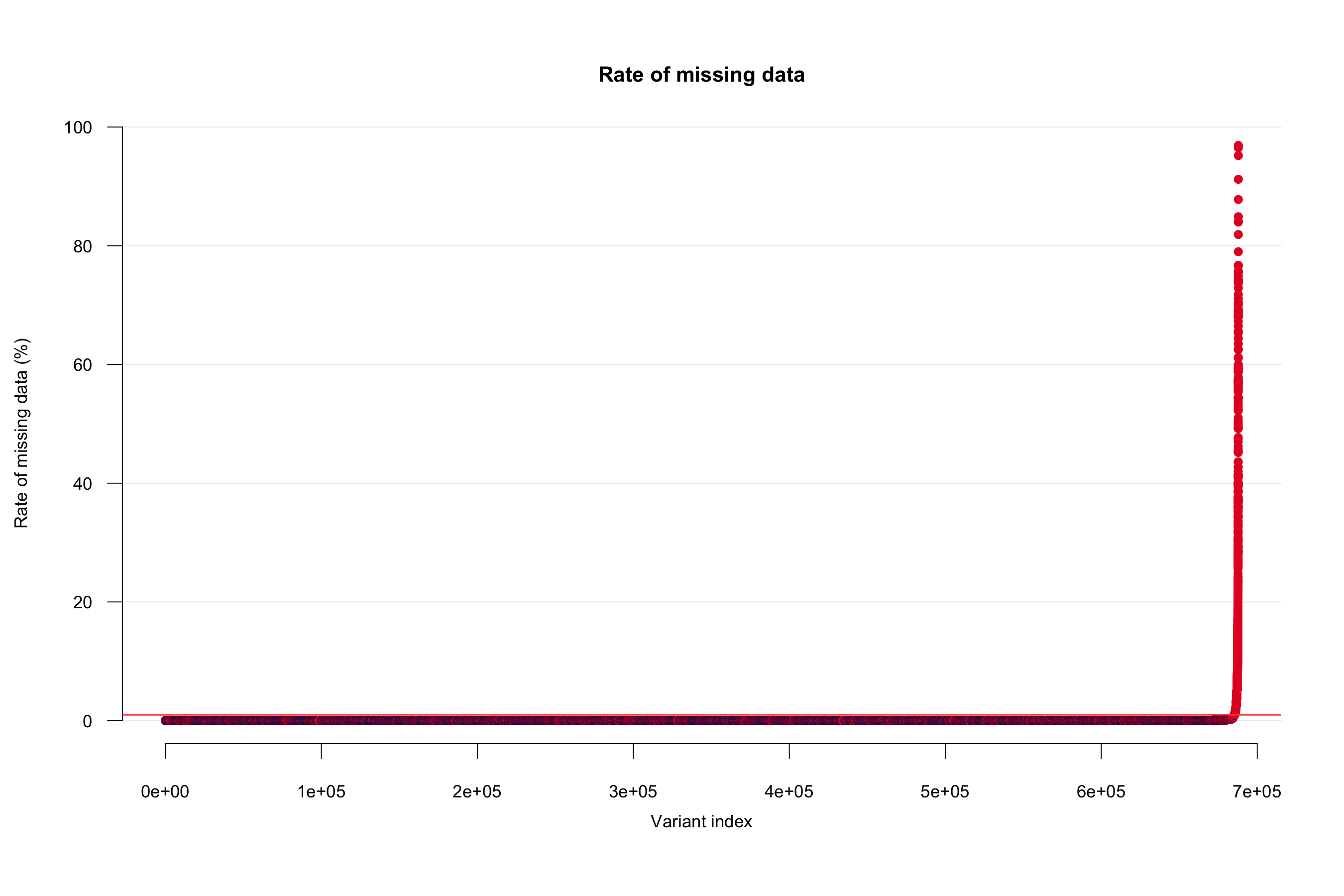** | **F** | **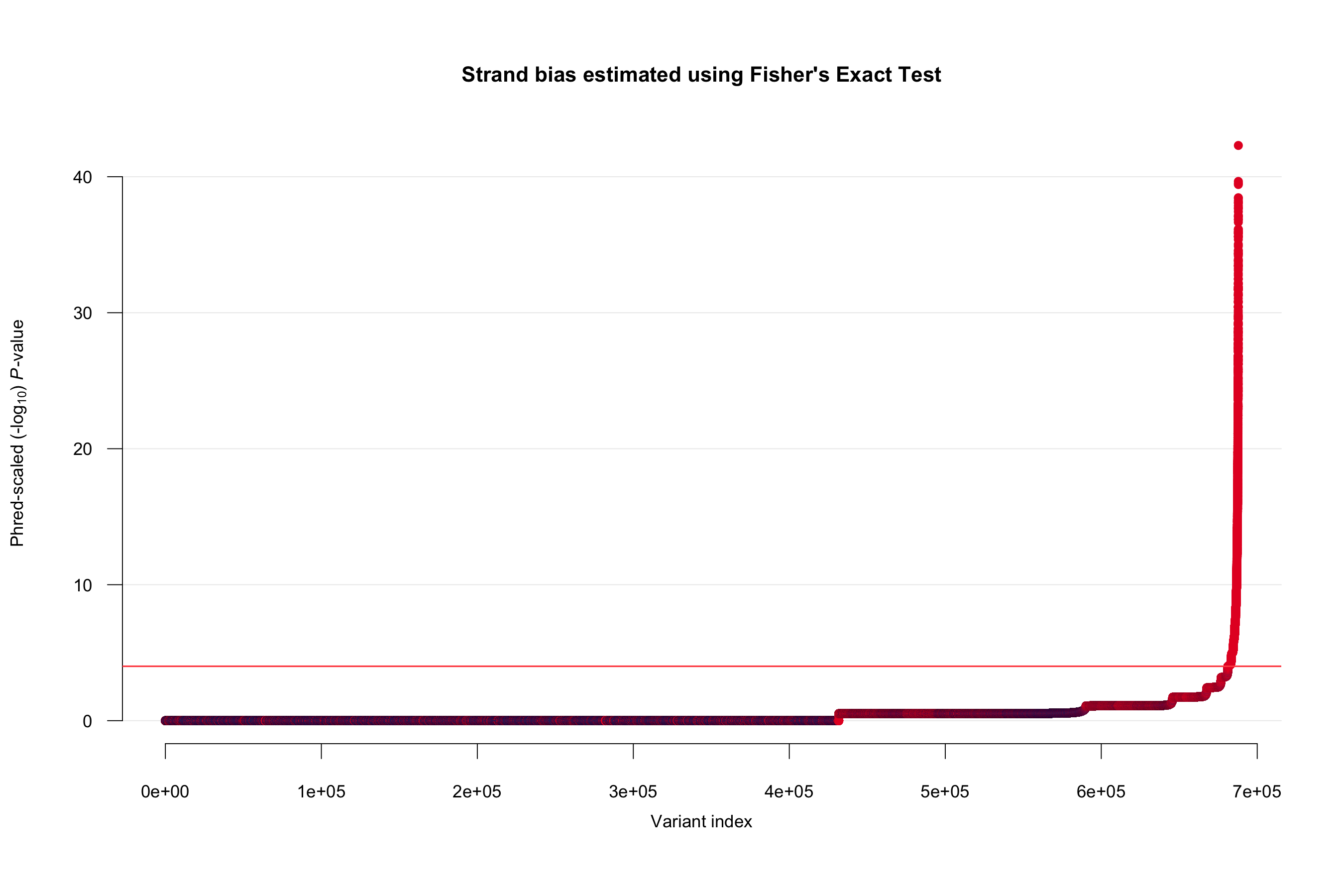** |

**Figure S2. Strict quality control based on 1kSWE quality parameters excluded a large proportion of variants.**

Panel (A)-(F) display sorted variants for six quality control parameters in the 1kSWE. Each data point corresponds to one variant. The horizontal red lines depict the cut-off levels for the given parameter. Red data points represent variants that were excluded from our dataset by any of the parameters. Since variant filters were applied in parallel, the number of red data points is equal in each of the six panels (A)-(F). Many variants failed in more than one parameter. Therefore, variants within the accepted range of for instance panel (A), could still be excluded and marked red based on the variant’s result in other parameters. Panels display (A) Rank sum test for hard-clipped bases on reference versus alternative reads, (B) Variant quality score log odds, (C) Total read depth across samples, (D) Variant call confidence normalized by depth, (E) Rate of missing data, and (F) Strand bias estimated using Fisher’s exact test.

| **A**  **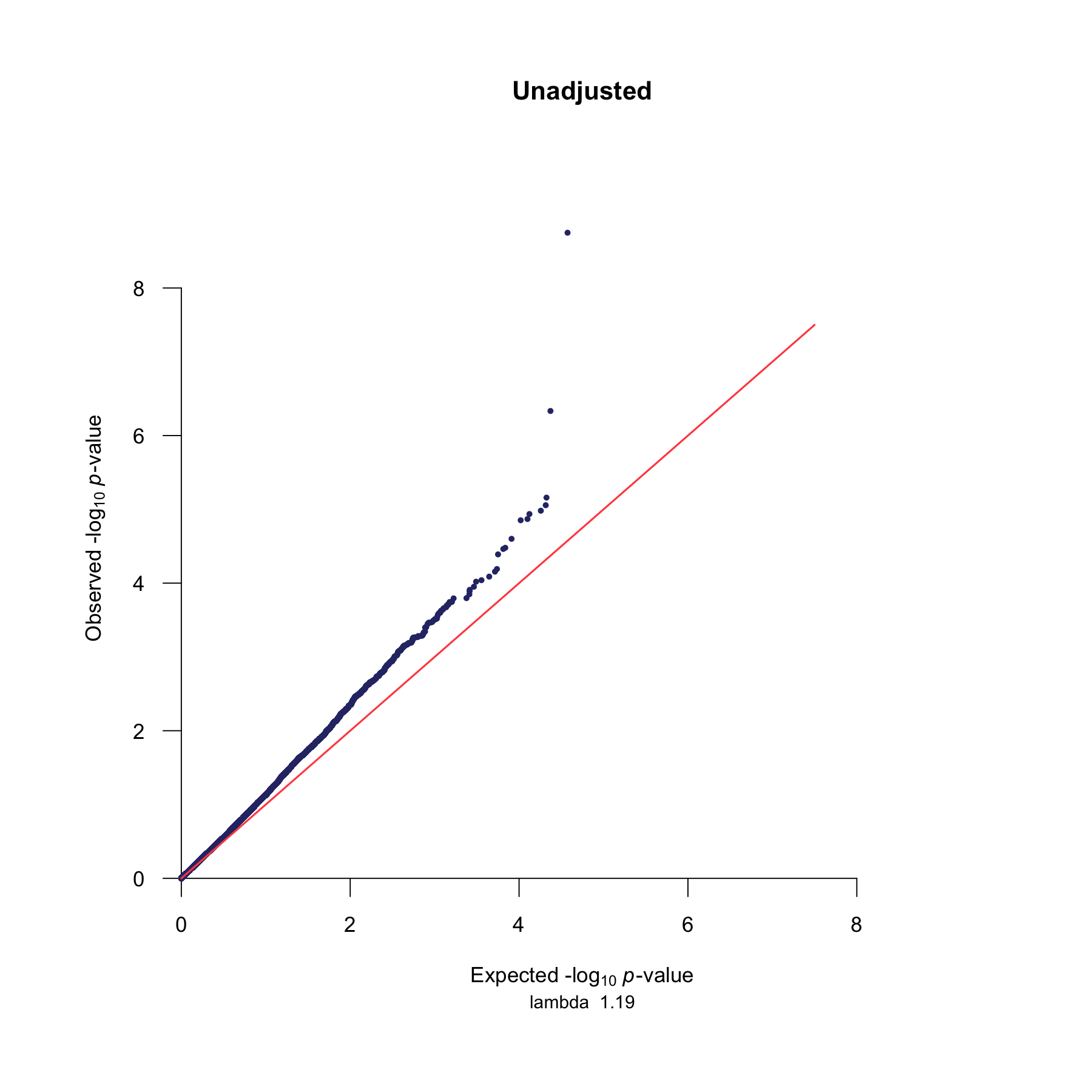** | **B**  **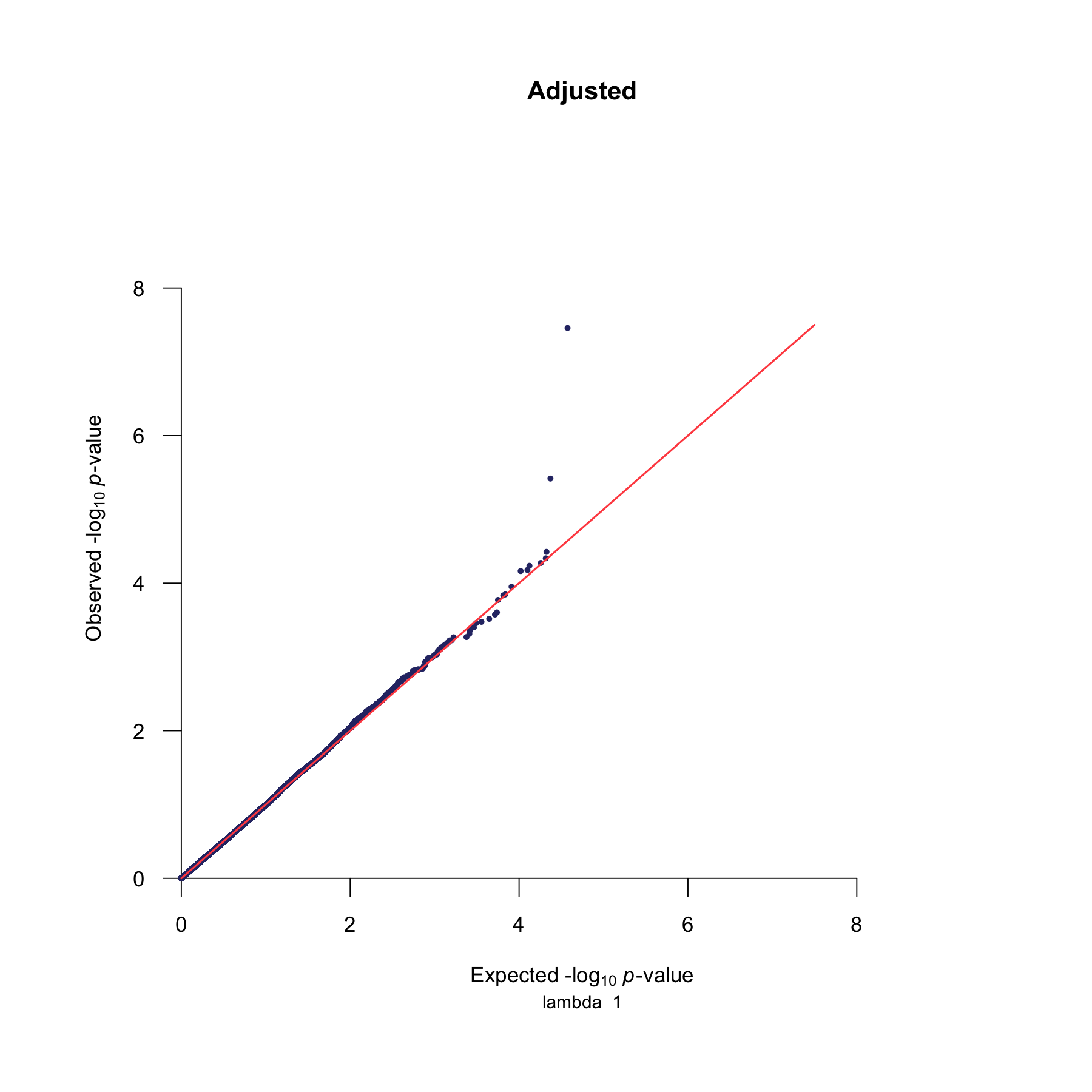** |
| --- | --- |

**Figure S3. Adjustment with genomic control reduced inflation before association testing.**

Quantile-quantile plots displaying observed P values versus random uniform distributions. Panel (A) shows *P* values from the crude Cochran-Armitage test statistic (λ = 1.19), whereas panel (B) shows *P* values after genomic control (λ = 1.00). Only adjusted *P* values were used for downstream analyses.

**
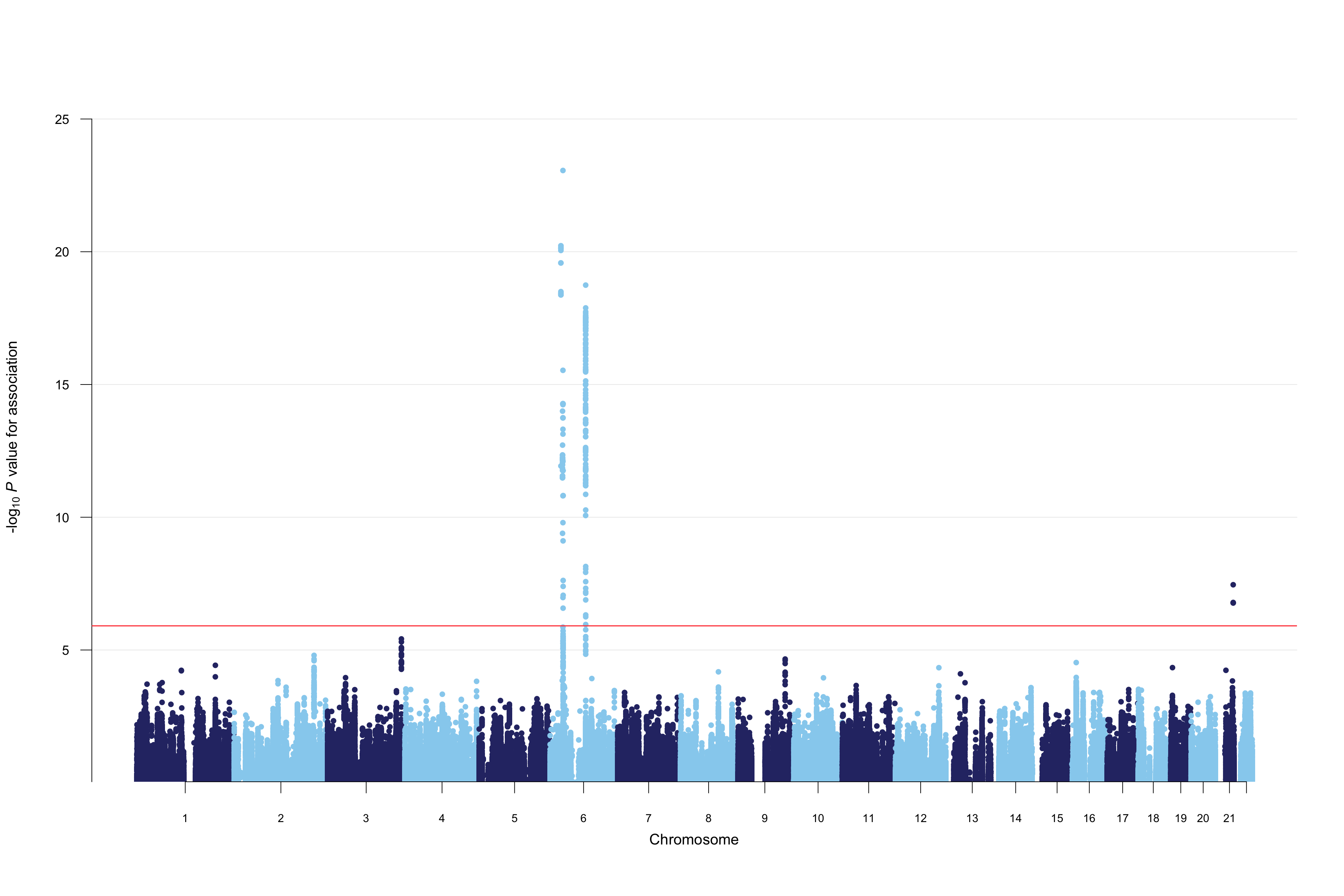
**

**Figure S4. Association between single-nucleotide polymorphisms and autoimmune Addison’s disease, exhibited as *P* values and genomic location.**

The association between SNPs and AAD, as calculated by Cochran-Armitage test for trend, is plotted as the negative log of the *P* value against chromosomal location. *P* values are adjusted with genomic control. The horizontal red line denotes the statistical significance level of 1.2 × 10^-6^.


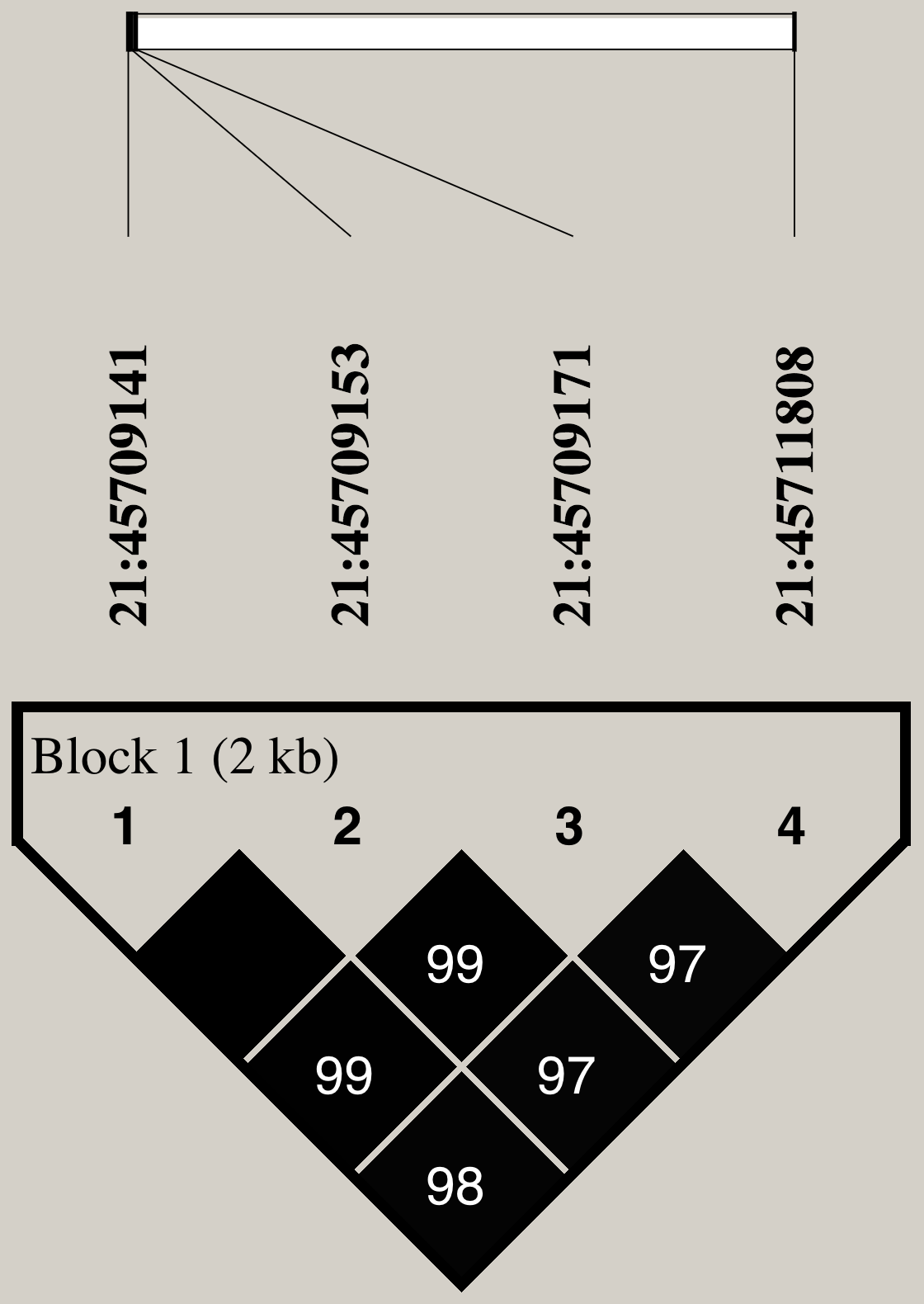


**Figure S5. Correlation between the associated SNPs visualized by Haploview.**

Colour scheme and LD values are set to ‘R-squared’, in Haploview v4.2. Genotypes from the 479 AAD cases and 1394 controls in the SAR-Seq study.


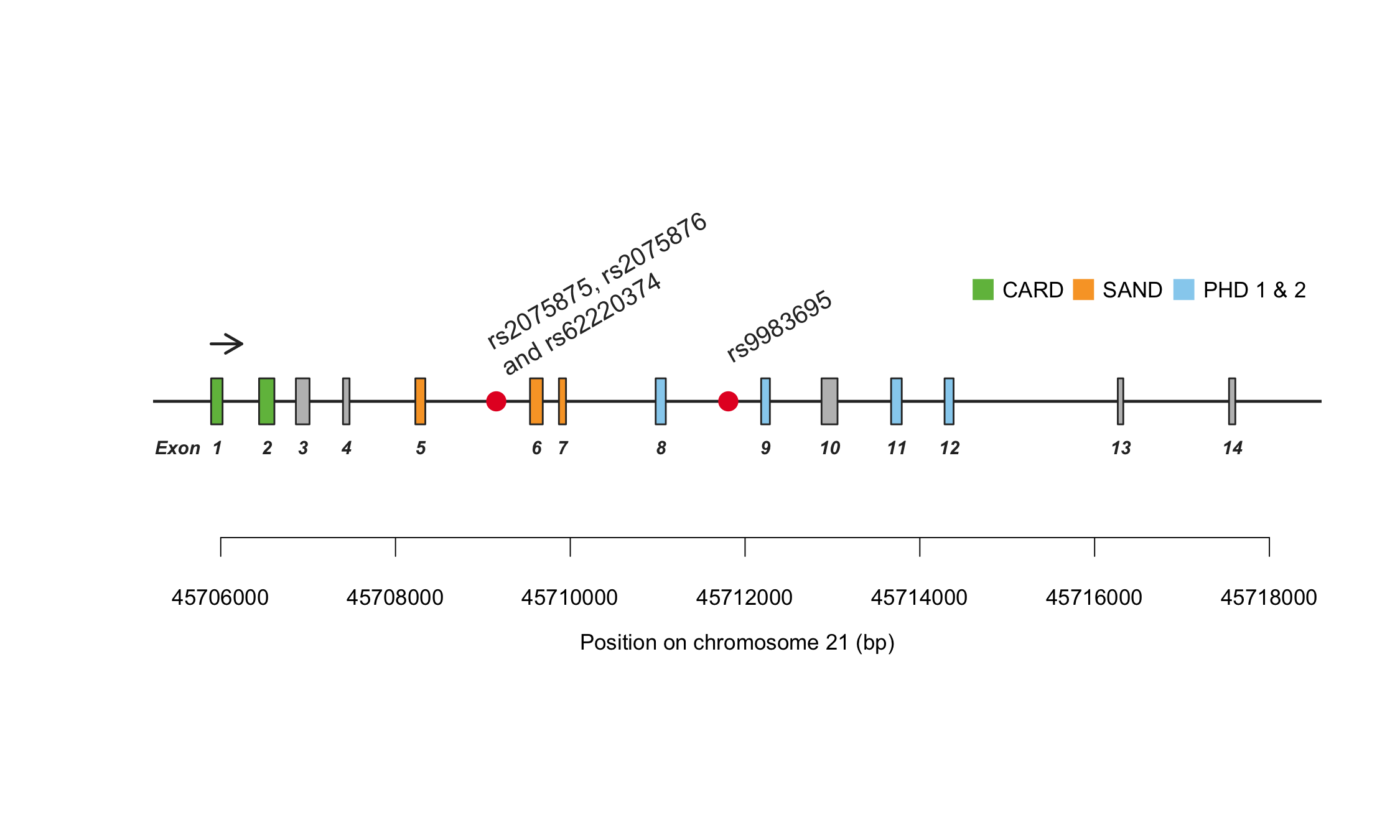


**Figure S6. A schematic view of the location of associated variants in the autoimmune regulator (*AIRE*) gene.**

Boxes indicate the size and position of the 14 exons in the main transcript (NM_000383). The positions of associated variants are marked with red points. Due to adjacent positions for three of the variants, their red points overlap and only two red points are distinguishable in the figure. The gene is transcribed from left to right, as indicated by the arrow. Although somewhat simplified, exon colours represent the corresponding domains of the translated protein. Gray exons have not been classified to any protein domain.

| **Table S2.** | | | | | | | | | | |
| --- | --- | --- | --- | --- | --- | --- | --- | --- | --- | --- |
|  |  |  |  |  | **Minor Allele Frequency** | | | |  |  |
| **Locus** | **Variant** | **Position** | **Function** | **Minor/Major Allele** | **SAR-Seq Cases** | **SAR-Seq Controls** | **1kSWE Controls** | **All Controls** | **OR** | ***P* value** |
| *AIRE* | rs2075875 | 45709141 | intronic | C/T | 0.04 | 0.10 | 0.10 | 0.10 | 0.40 (0.29-0.55) | 5.8 × 10^-7^ |
| *AIRE* | rs2075876 | 45709153 | intronic | A/G | 0.04 | 0.10 | 0.10 | 0.10 | 0.39 (0.28-0.55) | 5.5 × 10^-7^ |
| *AIRE* | rs62220374 | 45709171 | intronic | G/C | 0.04 | 0.10 | 0.10 | 0.10 | 0.40 (0.29-0.55) | 5.7 × 10^-7^ |
| *AIRE* | rs9983695 | 45711808 | intronic | C/T | 0.04 | 0.11 | 0.10 | 0.11 | 0.37 (0.27-0.52) | 1.4 × 10^-7^ |

|  |  |  | **Minor Allele Frequency in the 1000 genomes project populations** | | | |  | **AF in gnomAD** |  |
| --- | --- | --- | --- | --- | --- | --- | --- | --- | --- |
| **Locus** | **Variant** | **Position** | **AFR** | **AMR** | **ASN** | **EUR** |  | **EUR (Non-Finnish)** | **Imputed in our dataset** |
| *AIRE* | rs2075875 | 45709141 | 0.39 | 0.17 | 0.39 | 0.11 |  | 0.11 | No |
| *AIRE* | rs2075876 | 45709153 | 0.39 | 0.17 | 0.39 | 0.11 |  | 0.10 | No |
| *AIRE* | rs62220374 | 45709171 | 0.41 | 0.19 | 0.39 | 0.11 |  | 0.10 | No |
| *AIRE* | rs9983695 | 45711808 | 0.63 | 0.20 | 0.40 | 0.11 |  | 0.11 | No |

| **Locus** | **Variant** | **Position** | **DNAse Hypersensitivity** | **eQTL** | **Regulome Binding Evidence** | **Affected Motifs** | **GWAS Associations** |
| --- | --- | --- | --- | --- | --- | --- | --- |
| *AIRE* | rs2075875 | 45709141 | Fetal thymus | - | Minimal | BDP1, HNF4, RXRA | . |
| *AIRE* | rs2075876 | 45709153 | Fetal thymus | - | Minimal | LF-A1, NRSF | Rheumatoid arthritis (PMID 21505073) |
| *AIRE* | rs62220374 | 45709171 | Fetal thymus | - | Minimal | CTCF, HEY1, NRSF, Sin3Ak-20 | . |
| *AIRE* | rs9983695 | 45711808 | Breast | - | Minimal | Egr-1, GATA, Irf, Pax-5 | . |
| Abbreviations: SAR-Seq Swedish Addison Registry Sequencing Study; OR Odds Ratio; AFR African; AMR American; ASN Asian; EUR European; eQTL Expression quantitative trait locus; PMID PubMed ID. | | | | | | | |

| **Table S3. The number of risk alleles in cases and controls. Odds ratios and *P* values are calculated with the stratum 0-2 as reference in the Prism v6.0 software.** | | | | |
| --- | --- | --- | --- | --- |
| **Number of risk alleles** | **Cases** | **Controls** | **Odds ratio (95% CI)** | ***P* value** |
| 0-2 | 1 | 83 | (ref) | - |
| 3 | 7 | 157 | 3.7 (0.45-31) | 0.19 |
| 4 | 23 | 270 | 4.2 (0.55-32) | 0.13 |
| 5 | 54 | 348 | 13 (1.8-94) | 0.0013 |
| 6 | 122 | 279 | 36 (5.0-260) | < 0.0001 |
| 7 | 109 | 179 | 51 (6.9-370) | < 0.0001 |
| 8 | 95 | 58 | 140 (18-1000) | < 0.0001 |
| 9 | 44 | 18 | 200 (26-1600) | < 0.0001 |
| > 9 | 24 | 2 | 1000 (86-11000) | < 0.0001 |
|  |  |  |  |  |

| **Table S4. Gene-Gene interaction test for variants known to be associated to autoimmune Addison’s disease. Output from PLINK epistasis test. The constructed marker HLA-DRB1 represents any of the risk alleles 03:01, 04:04 and 04:03.** | | | | | | | |
| --- | --- | --- | --- | --- | --- | --- | --- |
| **CHR** | **SNP** | **N_SIG** | **N_TOT** | **PROP** | **BEST_CHISQ** | **BEST_CHR** | **BEST_SNP** |
| 1 | 1:114377568 | 0 | 7 | 0 | 1.048 | 2 | 2:204742934 |
| 2 | 2:204709349 | 0 | 7 | 0 | 1.556 | 21 | HLA-DRB1 |
| 2 | 2:204742934 | 0 | 7 | 0 | 1.464 | 16 | 16:11189148 |
| 2 | 2:204745003 | 0 | 7 | 0 | 2.815 | 16 | 16:11189148 |
| 6 | HLA-DRB1 | 0 | 7 | 0 | 1.784 | 2 | 2:204745003 |
| 6 | 6:90976609 | 0 | 7 | 0 | 2.386 | 21 | 21:45711808 |
| 16 | 16:11189148 | 0 | 7 | 0 | 3.296 | 21 | 21:45711808 |
| 21 | 21:45711808 | 0 | 7 | 0 | 3.296 | 16 | 16:11189148 |
| Abbreviations: CHR chromosome; SNP position/marker; N_SIG number of significant interactions; N_TOT number of investigated interactions, PROP proportion, BEST_CHISQ best chisquare statistic, BEST_CHR best interacting chromosome, BEST_SNP best interacting position/marker. | | | | | | | |

| **A** | **B** |
| --- | --- |
|  | 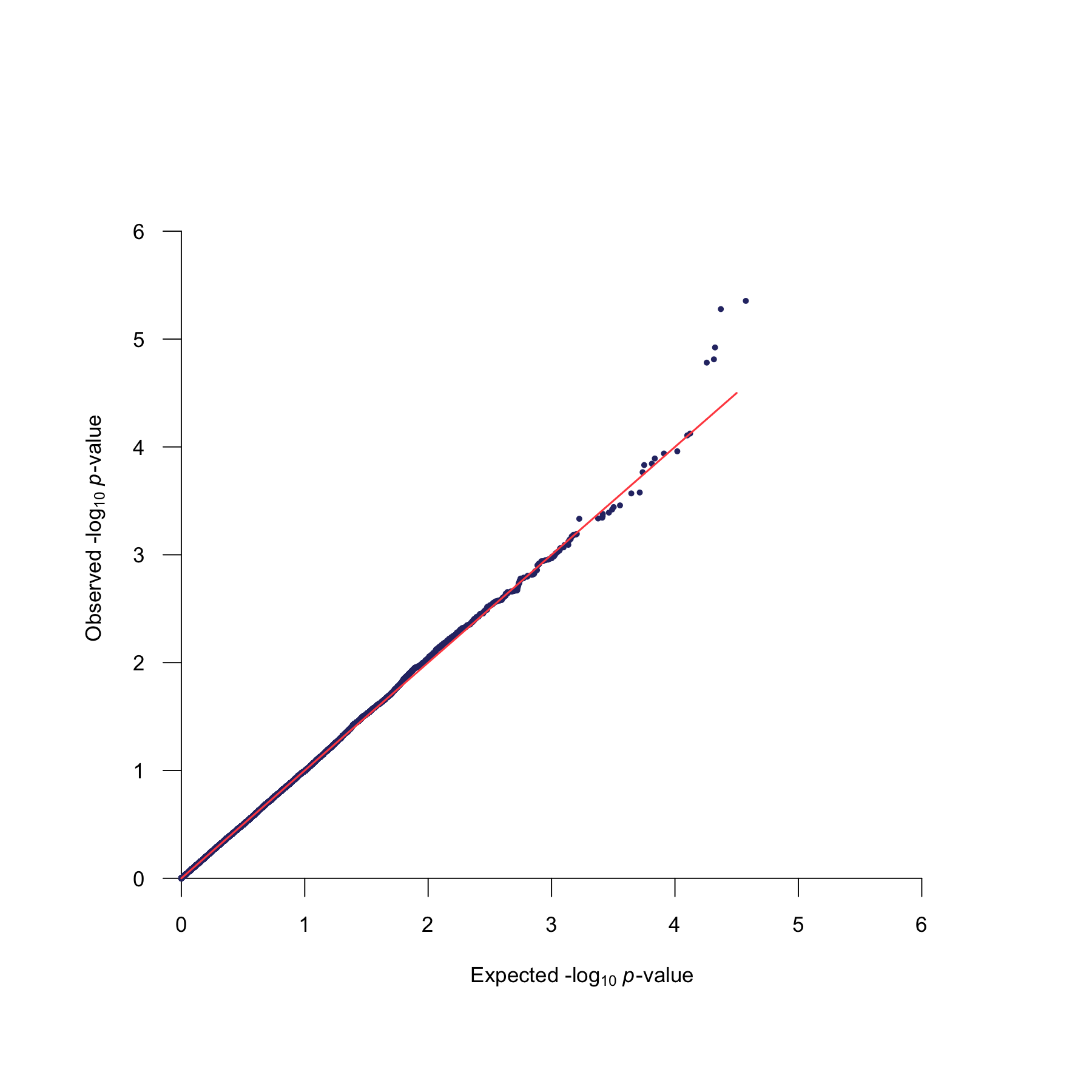 |

**Figure S7.**

**A) Age of onset plotted against the number of risk alleles at six selected loci.**

To assess whether an individual’s load of risk alleles can explain their age of disease onset, we stratified the cases by their number of risk alleles and fitted a linear regression model: Onset age = 43.9 – 1.39*x*, where *x* the number of additional risk alleles, is depicted by a dashed red line. On average, subjects with more than nine risk alleles acquired their AAD more than eight years earlier than subjects with less than five risk alleles (*P* = 0.018, 95% CI: -15.8 to -1.5) (Table below). However, the distribution within each stratum is wide and does not enable any individual predictions (R^2^ = 2.2%).

**B) Quantile-quantile plot displaying observed *P* values versus a random uniform distribution.**

Assuming that there is a genetic component influencing the age of disease onset, we associated SNPs to the quantitative trait within the 479 cases. SNPs were associated to age of onset in a linear regression model (PLINK --linear), using ten PCA components to adjust for population stratification (n=479). The plot shows that no single-nucleotide polymorphism exceeded our study-wide significance level of *P* < 1.2 × 10^-6^. The top SNP (rs1569495-C on chromosome 22, *P* = 5.0 × 10^-6^, MAF 0.2356) had a false discovery rate of 23%.

| **Table S5. Two sample t-test comparing average age of onset (years) in risk allele strata. The stratum with less than five risk alleles is used as a reference.** | | | | |
| --- | --- | --- | --- | --- |
| **Number of risk alleles** | **Difference in means** | **t (df)** | **95% CI** | ***P* value** |
| <5 | Ref. | - | - | - |
| 5 | -3.4 | 1.2 (83) | -9,045 to 2,258 | 0.24 |
| 6 | -1.7 | 0.59 (151) | -7,315 to 3,937 | 0,5539 |
| 7 | -7.0 | 2.7 (138) | -11,87 to -1,935 | 0,0068** |
| 8 | -5.7 | 2.1 (124) | -11,02 to -0,2848 | 0,0392* |
| 9 | -7.0 | 2.1 (73) | -13,64 to -0,4314 | 0,0371* |
| > 9 | -8.7 | 2.4 (53) | -15,81 to -1,542 | 0,0181* |
|  |  |  |  |  |

| **Table S6. Our results for single-nucleotide variants associated to Addison’s disease in previous studies. The columns with sample size calculations display the estimated number of cases needed to discover a novel risk allele given the allele frequencies and effect sizes in our study, a traditional GWAS cutoff (α = 5 ×10^-8^), power 80% and a ratio of cases and controls 1:1 (κ 1) and 1:5 (κ 5), respectively. The last column describes whether the variant has been imputed or not.** | | | | | | | | | | | | |
| --- | --- | --- | --- | --- | --- | --- | --- | --- | --- | --- | --- | --- |
|  |  |  | **Minor Allele Frequency** | |  | **Association** | |  | **Sample Size Calculation** | |  |  |
| **Locus** | **Variant** | **Chr:Position^1^** | **AAD-Seq Cases** | **All Controls** |  | **OR^2^** | ***P* value^3^** |  | **κ 1** | **κ 5** |  | **Imputed in our dataset** |
| *PTPN22* | rs2476601 | 1:114377568 | 0.18 | 0.12 |  | 1.52 (1.26-1.84) | 6.1×10^-5^ * |  | 3631 | 1965 |  | No |
| *CTLA4* | rs231806 | 2:204709349 | 0.30 | 0.36 |  | 0.74 (0.64-0.87) | 5.8×10^-4^ * |  | 4139 | 2566 |  | Yes |
| *CTLA4* | rs231775 | 2:204732714 | 0.53 | 0.45 |  | 1.37 (1.19-1.58) | 5.5×10^-5^ * |  | 3214 | 1924 |  | No |
| *CTLA4* | rs11571302 | 2:204742934 | 0.34 | 0.41 |  | 0.77 (0.66-0.89) | 1.6×10^-3^ * |  | 4806 | 2948 |  | Yes |
| *CTLA4* | rs7565213 | 2:204743409 | 0.34 | 0.41 |  | 0.77 (0.66-0.89) | 1.6×10^-3^ * |  | 4806 | 2948 |  | Yes |
| *CTLA4* | rs11571297 | 2:204745003 | 0.36 | 0.42 |  | 0.77 (0.66-0.90) | 2.4×10^-3^ * |  | 4859 | 2972 |  | Yes |
| *BACH2* | rs3757247 | 6:90957463 | 0.55 | 0.40 |  | 1.20 (1.04-1.38) | 3.4×10^-15^ * |  | 845 | 503 |  | Yes |
| *BACH2* | rs62408233 | 6:90976609 | 0.46 | 0.30 |  | 2.04 (1.77-2.36) | 1.8×10^-19^ * |  | 686 | 388 |  | No |
| *PD-L1* | rs1411262 | 9:5459419 | 0.22 | 0.25 |  | 0.85 (0.71-1.00) | 0.07 |  | 15975 | 9871 |  | No |
| *GATA3* | rs3802604 | 10:8102272 | 0.35 | 0.37 |  | 0.93 (0.80-1.08) | 0.37 |  | > 20 000 | > 20 000 |  | No |
| *GATA3* | rs569421 | 10:8108592 | 0.23 | 0.23 |  | 0.98 (0.83-1.16) | 0.86 |  | > 20 000 | > 20 000 |  | Yes |
| *CYP27B1* | rs4646536 | 12:58157988 | 0.35 | 0.37 |  | 0.94 (0.81-1.09) | 0.44 |  | > 20 000 | > 20 000 |  | No |
| *CYP27B1* | rs10877012 | 12:58162085 | 0.35 | 0.37 |  | 0.93 (0.80-1.07) | 0.34 |  | > 20 000 | > 20 000 |  | No |
| *CYP27B1* | rs703842 | 12:58162739 | 0.35 | 0.37 |  | 0.92 (0.79-1.06) | 0.30 |  | > 20 000 | > 20 000 |  | No |
| *CLEC16A* | rs12917716 | 16:11189148 | 0.35 | 0.41 |  | 0.78 (0.67-0.91) | 2.3×10^-3^ * |  | 5506 | 3368 |  | No |
| **1. All positions are given on reference genome hg19.**  **2. The odds ratio (OR) represents the effect of the minor allele on the odds of observing AAD, calculated across AAD-Seq Cases and all controls.**  **3. The presented *P* values are corrected with genomic control. * Asterisk marks statistical significance after Bonferroni correction for the 15 investigated SNVs (α = 3.3 ×10^-3^)** | | | | | | | | | | | | |
